# Recommendations for the individualised management of atypical hemolytic uremic syndrome in adults

**DOI:** 10.3389/fmed.2023.1264310

**Published:** 2023-12-01

**Authors:** Ana Ávila, Mercedes Cao, Mario Espinosa, Joaquín Manrique, Enrique Morales

**Affiliations:** ^1^Department of Nephrology, Hospital Universitario Dr. Peset, Valencia, Spain; ^2^Department of Nephrology, Hospital Universitario A Coruña, A Coruña, Spain; ^3^Department of Nephrology, Hospital Universitario Reina Sofía, Córdoba, Spain; ^4^Department of Nephrology, Complejo Hospitalario de Navarra, Pamplona, Spain; ^5^Department of Nephrology, Hospital Universitario 12 de Octubre, Madrid, Spain

**Keywords:** atypical hemolytic uremic syndrome, thrombotic microangiopathy, C5 inhibitor, early treatment initiation, treatment interruption

## Abstract

**Background:**

Despite significant advances in therapeutic management of atypical hemolytic uremic syndrome (aHUS), guidelines are not timely updated and achieving a consensus on management recommendations remains a topic of ongoing discussion.

**Methods:**

A Scientific Committee with five experts was set up. A literature review was conducted and publications addressing the classification of aHUS, patient profiles and therapeutic approach were selected. Recommendations were proposed at an initial meeting, evaluated through an online questionnaire and validated during a second meeting.

**Results:**

Patients with confirmed or clear suspicion of aHUS should be treated with C5 inhibitors within 24 h of the diagnosis or suspicion of aHUS. Treatment monitoring and the decision to interrupt treatment should be individualised according to the risk of relapse and each patient’s evolution. aHUS with a genetic variant or associated with pregnancy should be treated for at least 6–12 months; *de novo* aHUS associated with kidney transplant until renal function is recovered and genetic variants are ruled out; aHUS associated with malignant hypertension until genetic variants are ruled out; aHUS associated with non-kidney transplant, autoimmune diseases, infection-or drug-induced until the thrombotic microangiopathy is resolved. Patients with a high risk of relapse should be treated for longer than 6–12 months.

**Conclusion:**

These recommendations provides physicians who are not familiar with the disease with recommendations for the management of aHUS in adults. The experts who participated advocate early treatment, maintenance for at least 6–12 months and treatment interruption guided by genetic background, trigger factors, risk of relapse and evolution.

## Highlights

- Atypical hemolytic uremic syndrome (aHUS) is an ultrarare disease for which there is no clearly defined therapeutic approach in the current guidelines.
- This publication provides a set of recommendations for physicians involved in the clinical management of aHUS.
- aHUS should be treated with C5 inhibitors within 24 h of clinical suspicion, and treatment should be maintained for at least 6–12 months in specific patient profiles. Patients with a high risk of relapse might need longer treatment.
- Treatment interruption is feasible in selected patients with a low risk of relapse, as long as close follow-up is possible and C5 inhibitors are accessible in case of relapse.
- The therapeutic management of aHUS (duration of treatment, monitoring, interruption and follow-up) needs to be individualised according to the patient profiles presented in this publication.

## 1 Introduction

Atypical hemolytic uremic syndrome (aHUS) is an ultrarare complement-mediated thrombotic microangiopathy (TMA) characterised by nonimmune haemolytic anaemia, thrombocytopenia and acute kidney failure (1). aHUS is mainly caused by dysregulation of the alternative complement pathway (2, 3), resulting in inflammation, activation and damage to endothelial cells due to membrane-attack complex assembly (3). Mutations in complement pathway genes or the appearance of complement factor H (CFH) autoantibodies have been identified in 60–70% of patients (4, 5).

The signs and symptoms of aHUS are heterogeneous and complicate the diagnosis. Given its severity and rapid evolution, a differential diagnosis for other TMA causes is required. Determining ADAMTS13 activity and Shiga toxin testing are crucial to rule out thrombotic thrombocytopenic purpura (TTP) and Shiga toxin *E. coli* HUS (STEC-HUS), respectively. In adults, ADAMTS13 determination is particularly relevant for initiating complement component 5 (C5) inhibitors therapy and avoiding plasmapheresis. When immediate ADAMTS13 testing is unavailable, PLASMIC Score can help exclude TTP (a score > 6 is highly suggestive of TTP) (6, 7). Non-TTP or STEC-HUS cases should be diagnostically oriented towards aHUS (8, 9).

aHUS has historically been classified as primary when underlying abnormalities of the alternative complement pathway exist or when other causes traditionally linked to secondary aHUS are excluded. Secondary aHUS has been considered when it is precipitated by any of the well-known heterogeneous conditions or aetiological triggers, such as autoimmune diseases, malignancies, transplant, pregnancy, administration of certain drugs, or infections (8, 9). However, there is substantial overlap between these situations, and genetic defects in the alternative complement pathway can predispose to abnormal complement activation, while a secondary hit may propagate complement amplification (10). Additionally, genetic abnormalities may remain unidentified in carriers; indeed mutations go undetected in approximately 26% of familial aHUS cases (4). Therefore, there is an unmet need to identify patient profiles whose management can be more individualised.

aHUS was formerly associated with a poor prognosis in terms of mortality and renal function recovery after a first episode (4, 11, 12). The introduction of C5 inhibitors (eculizumab and ravulizumab) improved aHUS prognosis, leading to significant renal function recovery and TMA remission when administered during the acute phase (13–16).

Despite considerable progress in aHUS therapeutic management, guidelines are not timely updated, as C5 inhibitors are not universally considered first-line treatment (8, 9). Additionally, there is no consensus regarding which recommendations should be followed. Unequal access to diagnostic tests and to C5 inhibitors in many centres further complicates the development of clear and standardized recommendations. The START-aHUS (STrategy for monitoring and disease Risk-adapted Treatment of aHUS) consensus has been developed to address these gaps in aHUS therapeutic management, both generally and for specific patient profiles defined by expert consensus. This paper provides general and patient-specific recommendations regarding the initiation, duration and monitoring of discontinuation of aHUS treatment in adults.

## 2 Materials and methods

This study aimed to establish consensus recommendations for therapeutic management of aHUS patients by a group of highly experienced physicians in the field. A Scientific Committee comprising five nephrologists, all of whom were experts in aHUS management, was formed.

To gather relevant information, a literature review was conducted on the PubMed database^1^ within the predefined time period (2012–2022). The following keywords were used in the search: atypical hemolytic uremic syndrome, aHUS, thrombotic microangiopathy, TMA, complement, anti-complement therapies, eculizumab, and kidney disease. Selected publications addressing aHUS classification, including different patient profiles and dealing with its therapeutic approach were reviewed (Supplementary Table 1).

In March 2022, an online meeting involving all members of the Scientific Committee was convened to discuss recommendations to be issued to improve therapeutic management of aHUS patients throughout their journey: treatment initiation, monitoring, interruption, and post-interruption follow-up.

The recommendations were compiled in a questionnaire organised in two sections. The first section focused on the general recommendations, with participants providing their responses, personal comments and supporting bibliography. The second section covered recommendations on six specific patient profiles. The Scientific Committee completed the questionnaire in April 2022.

A second online meeting was held in May 2022 to reach consensus on the developed recommendations. This publication summarizes the recommendations discussed and agreed during this final meeting (Supplementary Figure 1).

## 3 Results

### 3.1 General recommendations on the therapeutic management of aHUS

**Figure 1:**
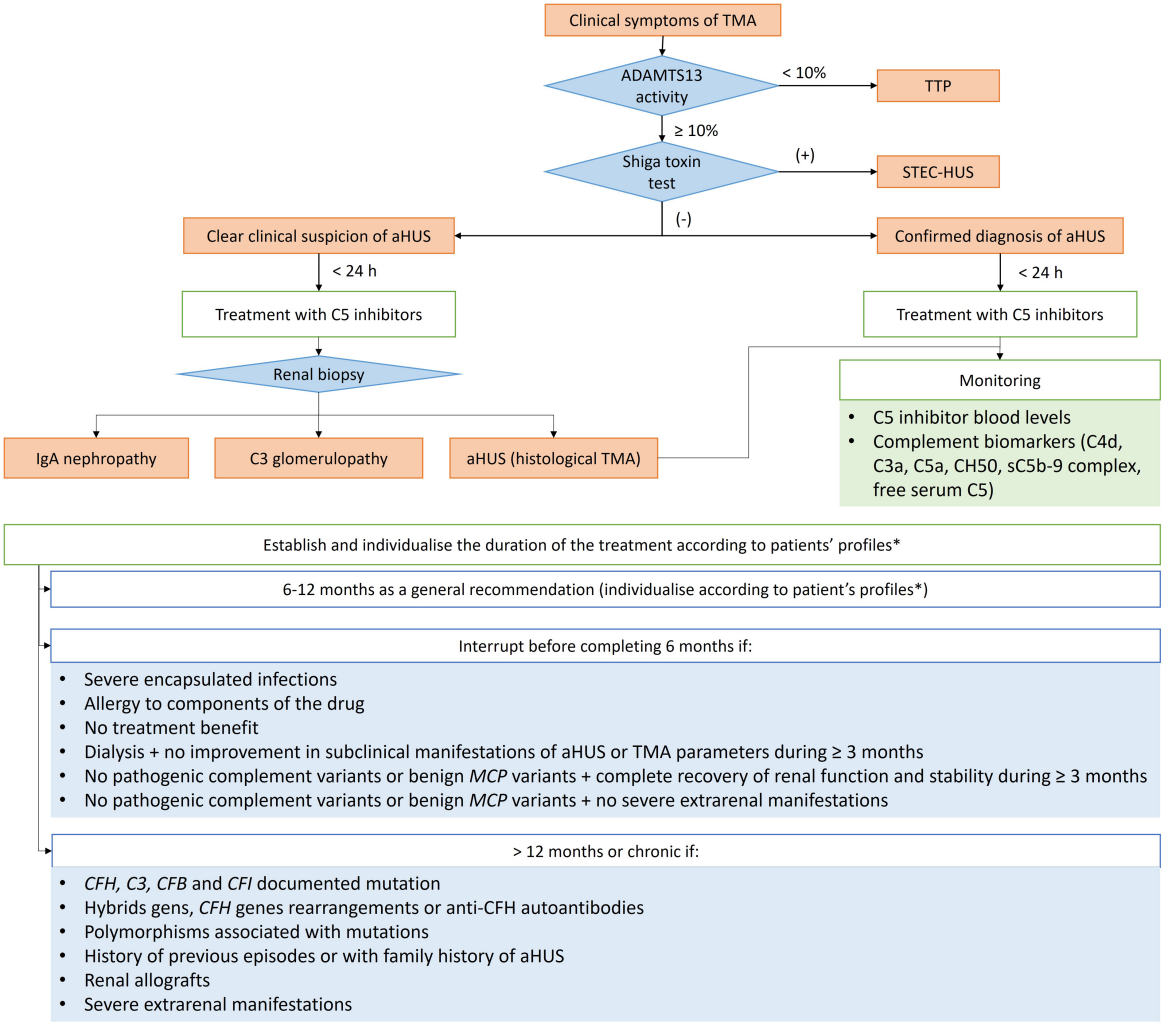
Diagram of the general treatment recommendations based on clinical evolution of adult patients with aHUS. aHUS, Atypical haemolytic uremic syndrome; *C3*, complement component 3; *CFB*, complement factor B; *CFH*, complement factor H; *CFI*, complement factor I; *MCP*, membrane-cofactor protein; MHT, Malignant hypertension; TMA, Thrombotic microangiopathy; TTP, thrombotic thrombocytopenic purpura; STEC-HUS, Shiga toxin *E. coli* HUS. ^*^Including the mutations identified, trigger, risk and evolution (see specific recommendations in Table 1).

Eculizumab and ravulizumab are anti-C5 monoclonal antibodies, and their efficacy and safety in patients with aHUS have stablished them as the first line therapies. However, their use continues to entail several challenges in terms of treatment initiation, duration, monitoring and interruption. In this regard, a set of recommendations are suggested and an algorithm summarising the general recommendations for the management of aHUS is shown (Figure 1).

**Table 1:** Recommendations for the management of the different aHUS patient profiles.

Step in the patient journey		aHUS profile					
		Genetic variants	MHT	Postpartum	*De novo* aHUS associated with kidney transplant	Non-kidney transplant or drug-induced	Autoimmune disease or infection
Diagnosis		Complete genetic study Functional study^a^ Kidney biopsy^b^	Complete genetic study^c^ Functional study Evaluation of the eye fundus Kidney biopsy^b^	Complete genetic study Functional study Kidney biopsy^b^	Complete genetic study Functional study Kidney biopsy^b^	Complete genetic study Functional study Kidney biopsy^b^	Complete genetic study Functional study Kidney biopsy^b^
Treatment duration^d^	Low risk of TMA recurrence	At least 6–12 months	At least 6–12 months Individualised until genetic variants are ruled out	At least 6–12 months	At least until recovery of renal function as long as no pathogenic variant is identified	Until resolution of TMA, as long as no pathogenic variant is identified	Until resolution of TMA, as long as no pathogenic variant is identified
	High risk of TMA recurrence	>12 months (even for life) if: *CFH*, *C3*, *CFB*, *CFI*, or *MCP* confirmed mutation; hybrid genes; *CFH* gene rearrangements or anti-CFH autoantibodies; polymorphisms combined with mutations					
Frequency of monitoring	Until discharge	Daily					
	From discharge until 6th month	Every 7–30 days (monthly in stabilised patients)					
Tests for monitoring	Haematological analysis^e^	x	x	x	x	x	x
	Renal analysis^f^	x	x	x	x	x	x
	Blood pressure	x	x	x	x	x	x
	Signs and symptoms of infection	x			x		
Factors to be evaluated for treatment interruption	Resolution of TMA					x	x
	Pathogenic variant	x	x	x	x	x	x
	Extrarenal manifestations	x	x	x	x		
	Clinical response^g^	x	x	x	x		
	Age^h^	x	x				
	Residual renal function	x					
	Transplant (renal or other)	x					
Post-interruption follow-up	Month 1	Monthly	Every 7–15 days	Every 7–15 days	Every 7–15 days	Every 7–15 days	Every 7–15 days
	Months 2–6	Monthly	Monthly	Monthly	Monthly	Monthly	Monthly
	Month 7 and onwards	Every 2–3 months	Every 2 months	Every 2 months	Every 2 months	Every 2 months	Every 2 months

aHUS, Atypical hemolytic uremic syndrome; *C3*, complement component 3 gene; *CFB*, complement factor B gene; *CFH*, complement factor H gene; *CFI*, complement factor I gene; *MCP*, membrane cofactor protein gene; MHT, Malignant hypertension; TMA, Thrombotic microangiopathy.

a Such as CH50 test.

b In cases in which the risk is acceptable in order to confirm the diagnosis (rule out other causes such as IgA-associated TMA or C3 glomerulopathy).

c Before the complete genetic study, is recommended to determine the aHUS risk score for patients with MHT. This score is not validated yet but represents a combination of features that helps to distinguish aHUS associated with MHT from other causes of MHT. The higher the score, the higher the probability of aHUS (17).

d In all aHUS profiles, treatment duration with C5 inhibitors should be stablished based on the mutation and the trigger factors of aHUS and must be individualised according to each patient’s risk and evolution.

e Including haemolysis parameters.

f Including proteinuria, urine sediment and renal function.

g Global clinical response is (a) a > 50% decrease in serum creatinine concentration and urinary protein-creatinine ratio (UPCR) and serum albumin concentration > 3 g/dl or (b) the persistence of normal serum creatinine concentration (±10%) and albumin concentration > 3 g/dl and normalization of UPCR (<150 mg/g). Partial clinical response is (a) < 50% decrease in serum creatinine concentration, albumin concentration > 3 g/dl, and >50% decrease in UPCR; or (b) the persistence of normal serum creatinine concentration (±10%), albumin concentration > 3 g/dl, and >50% decrease in UPCR.

h Treatment should be maintained in pediatric patients.

#### 3.1.1 Early initiation of treatment

- C5 inhibitors should be the first-line treatment of aHUS, particularly to reduce the need for dialysis and ICU admission time.
- Patients with a confirmed diagnosis (patients with relapsing aHUS or a confirmed family history of aHUS) must be treated within 24 h of the clinical suspicion.
- In the presence of a clear clinical suspicion of aHUS diagnosis, early initiation of treatment with a C5 inhibitor is recommended (as soon as possible and ideally within 24 h of clinical suspicion). In centres with limited C5 inhibitors access, initiate plasmapheresis until obtaining the complement inhibitor.

#### 3.1.2 Duration of treatment

- Overall, C5 inhibitor treatment should be maintained for at least 6 to 12 months.
- The minimum 6-month period must include at least 3 months of treatment after the normalisation of serum creatinine or the stabilisation of renal function (18).
- The duration of treatment with C5 inhibitors, as well as when it should be interrupted, depends on the mutation and the trigger factors of aHUS and must be individualised according to each patient’s risk and evolution.
- Treatment interruption should be considered in specific patient profiles before they have completed 6 months of treatment:
  ‒ In patients with *de novo* TMA after kidney transplant, maintain C5 inhibitor treatment at least until recovery of renal function and, in case of suspected mutation, at least until the results of the genetic study are available. In some cases, recovery of renal function may be rapid and require few treatment doses.
  ‒ Development of severe encapsulated infections.
  ‒ Allergy to any component of the drug.
  ‒ Lack of treatment benefit.
  ‒ Dialysis (renal replacement therapy) without worsening of subclinical manifestations of aHUS or without improvement in TMA parameters during the last 3 months of treatment or more.
  ‒ No pathogenic complement variants or benign variants of the membrane-cofactor protein (MCP) type, provided that:
    - A complete recovery of renal function and stability over the last 3 months is achieved, or
    - No severe extrarenal manifestations are observed.

#### 3.1.3 Treatment monitoring

- A renal biopsy should be performed as soon as possible to confirm the diagnosis (rule out other causes such as IgA-associated TMA or C3 glomerulopathy) in cases in which the risk is acceptable and in any aHUS profile.
- The identification of complement biomarkers by immunohistochemistry in a kidney biopsy can help to understand the pathogenesis of aHUS beyond the information provided by proteinuria, albuminuria, renal function and hypertension data.
- The use of biomarkers for monitoring is currently difficult. The combination of blood C5 inhibitor levels with a complement biomarker (C4d, C3a, C5a, and CH50) or plasma levels of sC5b-9 complex could provide relevant information (19).
- Despite not being specific biomarkers for aHUS, complement-specific urine levels, such as sC5-9 and factor Ba, are significantly associated with kidney disfunction, suggesting a potential prognostic utility in the management of complement-mediated TMA, including the assessment and prediction of response to anti-C5 therapy (20, 21). Also, the C5b-9 endothelial deposition assay enhance our ability to monitor disease activity and individualize therapy (22, 23).
- Biomarkers should be widely available in routine clinical practice in the future.
- Further research is needed to identify the optimal biomarkers for monitoring treatment with either eculizumab or ravulizumab.

#### 3.1.4 Post-treatment interruption follow-up

- Treatment interruption requires close patient monitoring and immediate access to treatment in case of documented recurrence (18).
- Patients must be informed about and trained in the warning signs and symptoms:

‒ Periodic determination of blood pressure.
‒ Periodic examination of changes in urine colour and the use of urine strips.
‒ Signs and symptoms of infection.

### 3.2 Recommendations on the therapeutic management of specific profiles of patients with aHUS

Distinguishing primary from secondary aHUS is still a challenge in many cases, especially when genetic variants are not detected or when they overlap with one of the triggering conditions of secondary aHUS. A classification based on easily-defined patient profiles was an important objective and considered to be helpful throughout the START project.

Six patient profiles were defined:

- aHUS associated with genetic variant of complement.
- aHUS associated with malignant hypertension (MHT).
- aHUS associated with pregnancy (P-aHUS).
- aHUS associated with *de novo* TMA after kidney transplant.
- aHUS associated with solid-organ non-kidney transplant or drug-induced aHUS (after a non-kidney transplant, aHUS is generally induced by drugs such as mTOR inhibitors or calcineurin inhibitors).
- aHUS associated with autoimmune disease or with infection.

The corresponding recommendations regarding treatment duration, monitoring, interruption and post-interruption follow-up were agreed on by the experts (Table 1; Supplementary Figure 2).

TMA have been detected in patients with MHT-associated aHUS, in which MHT is more a clinical manifestation of aHUS than a trigger. Suspicion of TMA must be even greater in young patients with severe acute kidney injury, without apparent causes of hypertension and with renal function that does not improve despite blood pressure control (24). Similarly, the presence of pathogenic variants in complement genes has been reported in patients with hypertension-induced TMA (25). MHT is the most severe form of high blood pressure and may entail many complications and has a poor prognosis (26). Moreover, a significant number of patients with MHT-associated aHUS may present complement-related endothelium damage, although mutations are not always identified. Consequently, the recommendation is to give C5 inhibitors until the presence of a pathogenic genetic variant has been excluded.

P-aHUS reportedly affects in 1 in 25,000 pregnancies (27) and occurs in 20% of women with aHUS (28). P-aHUS is associated with high mortality, morbidity and several consequences beyond the initial presentation. The risk of developing TMA is specially high during postpartum (29). P-aHUS is also commonly linked to known complement pathogenic mutations (about 50%) (29), and even in cases not associated with any of them, there must be an unidentifiable genetic component. Therefore, a complete genetic and functional study of the complement is highly recommended. The functional study, such as CH50 test (also called CH100), measures the amount or activity of all major complement system proteins, being C3 and C4 the most commonly analysed. Low CH 50 means different things: an *in vivo* consumption of complement proteins due to hyperactivation, deficiency of complement components or treatment with a complement inhibitor. A test showing abnormal levels or reduced/absent activity of those complement proteins, should raise suspicion of autoimmune disease or another serious health problem. Since both patients with P-aHUS receiving eculizumab (29–31) and those receiving ravulizumab (32) show excellent renal response, regardless of the presence of inherited complement abnormalities, they are all candidates for treatment with C5 inhibitors.

Kidney transplant is also a strong trigger for new-onset or recurrent aHUS (33). Mortality and recurrence rates range from 60 to 90% within the first year of onset (34, 35). To prevent recurrence after a kidney transplant, the use of prophylactic measures is recommended, except in patients considered to have a low risk of recurrence. Prophylaxis with C5 inhibitors should be started on the day of transplantation in patients with moderate-high recurrence risk due to potential for limited recovery of function in renal grafts (9).

Other profiles for which recommendations throughout the patient journey have been addressed in this work include: patients with aHUS associated with non-kidney transplant (mostly linked to immunosuppressive treatment); and patients with aHUS triggered by an autoimmune disease or an infection (see Table 1).

Early initiation of treatment with C5 inhibitors leads to improved renal and extrarenal outcomes. It also leads to less time in the ICU, less dialysis, fewer kidney transplants and lower hospitalisation costs (36, 37). Although it is currently difficult to confirm the diagnosis in advance and to begin treatment with certainty, early initiation is always recommended in case of clinical suspicion of aHUS. In this regard, there is currently a gap in the complete and rapid determination of genetic variants to initiate treatment with a clear diagnosis, which is also hampered by a lack of resources in certain centres and hospitals. Future investment of resources in this field must therefore be prioritised.

The need for continued eculizumab treatment has been a matter of debate. The available evidence supports continued treatment with C5 inhibitors for at least 6 months. A clinical benefit in TMA-defining parameters is still obtained beyond 6 months of treatment with C5 inhibitors. After 6 months, they also continue to prevent kidney disease progression (15, 38, 39). Additionally, extrarenal symptoms can persist for 6 months after the diagnosis of aHUS (8, 40–42). Some authors, such as the Dutch group that conducted the CUREiHUS study, emphasized the feasibility of interrupting eculizumab after 3 months in well-defined pediatric and adult patients with aHUS in native kidneys (43). Nevertheless, the safety of eculizumab interruption has been questioned in another study, which reported a 50% resumption of treatment after eculizumab interruption and a decline kidney function over time after discontinuation (44). Therefore, treatment duration must be individualised according to patient’s risk and evolution.

The interruption of C5 inhibitor treatment is feasible, although there are several considerations. First, relapses occur in 20–35% of patients at a median of 3 months after treatment cessation, and 90% of all relapses occur within 1 year of discontinuation (18). The presence of extrarenal manifestations prior to treatment, pathogenic variants or a family history of aHUS have been described as risk factors for TMA relapse (43). Other factors include CFH autoantibodies, paediatric onset, multiple TMA episodes, pregnancy, kidney transplant or impaired renal function (4, 18, 44–47). Mutations in *CFH*, *C3*, *CFB*, and *CFI* are also associated with risk of recurrence (18). Consequently, thorough risk assessment is crucial, and interruption should be avoided in patients presenting such characteristics. Second, interruption should be discussed extensively with each patient, who must be informed about the potential benefits and risks. Both physician and patient should be aware of a potential aHUS relapse (patient education for early detection of TMA symptoms), especially in the first year after treatment interruption, potentially triggering events (mainly infections), clinically relevant increases in serum creatinine and/or hematuria and/or proteinuria. Lastly, C5 inhibitors should always be available to immediately resume treatment in case of relapse.

Present recommendations are centred on aHUS management in adults, and do not address paediatric profiles such as diacylglycerol kinase-ε (DGKε) deficiency. As for aHUS associated with DGKε, which occurs in children under 1 year old, the mechanism behind endothelial damage remains unknown, it has been suggested that TMA in these patients may occur independently of complement dysregulation (48, 49). Anti-CFH autoantibodies have been described in sporadic forms of aHUS. These patients may respond to plasmapheresis and also benefit from immunosuppressive treatment. Until recovery of the antibody titers below 250 AU/ml, complement blockade treatment effectively halts TMA damaged and injury to target organs (50, 51).

## 4 Discussion

The START consensus provides expert-based recommendations which is intended to serve as a guide for other physicians with less experience in managing suspected aHUS cases, covering the entire patients journey from clinical suspicion through post-treatment interruption follow-up. Nevertheless, effective aHUS patient care relies on individualisation and adaptation of these general recommendations to specific patient profiles.

A correct and rapid identification of the specific profiles of aHUS patients would facilitate a more individualised therapeutic management, and thus help to avoid the progression of TMA. This consensus presents six patient profiles in accordance with their triggers, suggesting a differential approach for each one. Nevertheless, the presence of a pathogenic variant yet to be identified or defined in patients who meet these profiles should never be ruled out, particularly in aHUS associated with MHT or P-aHUS. Some biomarkers that may show utility in the prognosis of complement-mediated TMA, including their potential for measuring and predicting response to anti-C5 therapy, have been described (20). The identification of low levels of C3, CH50, AH50 and complement factor B along with increased levels of C5a, C5b-9 and factor Bb, could be indicative of aHUS; although the data available is unconclusive (52). Future research into the classification of patient profiles is relevant to improve the individualisation of therapeutic management and clinical response.

Hitherto, the different profiles of patients with aHUS were poorly defined and still conformed to the primary and secondary aHUS classification. The appearance of pathogenic variants or, in their absence, the factors that trigger TMA, were the prevailing defining variables. However, genetic or acquired defects in complement regulation and trigger factors seem to converge in most cases. Complement-related genetic mutations have been found in at least 50% of patients with aHUS (4, 8, 53). Similarly, the penetrance of aHUS in complement mutation carriers is approximately 50% (8). In some cases, mutations not related to the complement pathway, such as DGKε, have been described (49, 54).

Access to diagnostic tests for aHUS and C5 inhibitors treatment still are unequal and faces challenges. In general, ready access to C5 inhibitors should be available to avoid plasmapheresis after a suspected diagnosis of aHUS. Other complement-inhibiting therapies are currently under development, more data are needed, but in the future, they may emerge as an intriguing alternative in the treatment of aHUS. Similarly, treatment monitoring tools are not universally accessible in all hospitals. Thus, the implementation of the standardised recommendations offered in this document is crucial if the prognosis of all patients with aHUS is to be improved, regardless of the hospital or region where they are treated.

## 5 Conclusion

The START consensus provides a set of recommendations for the management of patients with aHUS based on the early initiation of C5 inhibitors, a minimum duration of 6–12 months and an evaluation of the suitability of interruption depending on genetic background, trigger factors and the evolution of each patient.

## Data availability statement

The original contributions presented in the study are included in the article/Supplementary material, further inquiries can be directed to the corresponding author.

## Author contributions

AÁ: Writing – original draft, Writing – review & editing. MC: Writing – original draft, Writing – review & editing. ME: Writing – original draft, Writing – review & editing. JM: Writing – original draft, Writing – review & editing. EM: Writing – original draft, Writing – review & editing.

## Footnotes

^1^https://pubmed.ncbi.nlm.nih.gov/

## References

[1] Fakhouri F Zuber J Frémeaux-Bacchi V Loirat C. Haemolytic uraemic syndrome. Lancet. (2017) 390:681–96. doi: 10.1016/S0140-6736(17)30062-4 28242109

[2] Loirat C Frémeaux-Bacchi V. Atypical hemolytic uremic syndrome. Orphanet J Rare Dis. (2011) 6:60–30. doi: 10.1186/1750-1172-6-60 21902819 PMC3198674

[3] Sakari JT. HUS and atypical HUS. Blood. (2017) 129:2847–56. doi: 10.1182/blood-2016-11-709865 28416508 PMC5445567

[4] Noris M Caprioli J Bresin E Mossali C Pianetti G Gamba S. Relative role of genetic complement abnormalities in sporadic and familial aHUS and their impact on clinical phenotype. Clin J Am Soc Nephrol. (2010) 5:1844–59. doi: 10.2215/CJN.02210310, PMID: 20595690 PMC2974386

[5] Fremeaux-Bacchi V Fakhouri F Garnier A Bienaimé F Dragon-Durey MA Ngo S. Genetics and outcome of atypical hemolytic uremic syndrome: a nationwide French series comparing children and adults. Clin J Am Soc Nephrol. (2013) 8:554–62. doi: 10.2215/CJN.04760512, PMID: 23307876 PMC3613948

[6] Uriol M Rivera U Bernabeu A Iii J Ernst F Comas A. (2022). PLASMIC score to aid diagnosis of aHUS: a post-hoc analysis of data from C5 inhibitor trials. American Society of Nephrology.

[7] Bendapudi PK Hurwitz S Fry A Marques MB Waldo SW Li A. Derivation and external validation of the PLASMIC score for rapid assessment of adults with thrombotic microangiopathies: a cohort study. Lancet Haematol. (2017) 4:e157–64. doi: 10.1016/S2352-3026(17)30026-1, PMID: 28259520

[8] Campistol JM Arias M Ariceta G Blasco M Espinosa L Espinosa M. Actualización en síndrome hemolítico urémico atípico: diagnóstico y tratamiento. Documento de consenso. Nefrologia. (2015) 35:421–47. doi: 10.1016/j.nefro.2015.07.005 26456110

[9] Goodship THJ Cook HT Fakhouri F Fervenza FC Frémeaux-Bacchi V Kavanagh D. Atypical hemolytic uremic syndrome and C3 glomerulopathy: conclusions from a “kidney disease: improving global outcomes” (KDIGO) controversies conference. Kidney Int. (2017) 91:539–51. doi: 10.1016/j.kint.2016.10.005, PMID: 27989322

[10] Gavriilaki E Brodsky RA. Complementopathies and precision medicine. J Clin Invest. (2020) 130:2152–63. doi: 10.1172/JCI136094, PMID: 32310222 PMC7190924

[11] Caprioli J Noris M Brioschi S Pianetti G Castelletti F Bettinaglio P. Genetics of HUS: the impact of MCP, CFH, and IF mutations on clinical presentation, response to treatment, and outcome. Blood. (2006) 108:1267–79. doi: 10.1182/blood-2005-10-007252, PMID: 16621965 PMC1895874

[12] Sellier-Leclerc AL Fremeaux-Bacchi V Dragon-Durey MA Macher MA Niaudet P Guest G. Differential impact of complement mutations on clinical characteristics in atypical hemolytic uremic syndrome. J Am Soc Nephrol. (2007) 18:2392–400. doi: 10.1681/ASN.2006080811, PMID: 17599974

[13] Legendre CM Licht C Muus P Greenbaum LA Babu S Bedrosian C. Terminal complement inhibitor eculizumab in atypical hemolytic-uremic syndrome. N Engl J Med. (2013) 368:2169–81. doi: 10.1056/NEJMoa1208981 23738544

[14] Greenbaum LA Fila M Ardissino G Al-Akash SI Evans J Henning P. Eculizumab is a safe and effective treatment in pediatric patients with atypical hemolytic uremic syndrome. Kidney Int. (2016) 89:701–11. doi: 10.1016/j.kint.2015.11.026, PMID: 26880462

[15] Rondeau E Scully M Ariceta G Barbour T Cataland S Heyne N. The long-acting C5 inhibitor, Ravulizumab, is effective and safe in adult patients with atypical hemolytic uremic syndrome naïve to complement inhibitor treatment. Kidney Int. (2020) 97:1287–96. doi: 10.1016/j.kint.2020.01.035, PMID: 32299680

[16] Ariceta G Dixon BP Kim SH Kapur G Mauch T Ortiz S. The long-acting C5 inhibitor, ravulizumab, is effective and safe in pediatric patients with atypical hemolytic uremic syndrome naïve to complement inhibitor treatment. Kidney Int. (2021) 100:225–37. doi: 10.1016/j.kint.2020.10.046, PMID: 33307104

[17] Fakhouri F Schwotzer N Frémeaux-Bacchi V. How I diagnose and treat atypical hemolytic uremic syndrome. Blood. (2023) 141:984–95. doi: 10.1182/blood.2022017860, PMID: 36322940

[18] Laurence J. Defining treatment duration in atypical hemolytic uremic syndrome in adults: a clinical and pathological approach. Clin Adv Hematol Oncol. (2020) 18:221–30. PMID: 32628650

[19] Palomo M Blasco M Molina P Lozano M Praga M Torramade-Moix S. Complement activation and thrombotic microangiopathies. Clin J Am Soc Nephrol. (2019) 14:1719–32. doi: 10.2215/CJN.05830519, PMID: 31694864 PMC6895490

[20] Cammett TJ Garlo K Millman EE Rice K Toste CM Faas SJ. Exploratory prognostic biomarkers of complement-mediated thrombotic microangiopathy (CM-TMA) in adults with atypical hemolytic uremic syndrome (aHUS): analysis of a phase III study of Ravulizumab. Mol Diagn Ther. (2023) 27:61–74. doi: 10.1007/s40291-022-00620-3, PMID: 36329366 PMC9813049

[21] Cofiell R Kukreja A Bedard K Yan Y Mickle AP Ogawa M. Eculizumab reduces complement activation, inflammation, endothelial damage, thrombosis, and renal injury markers in aHUS. Blood. (2015) 125:3253–62. doi: 10.1182/blood-2014-09-600411, PMID: 25833956 PMC4449039

[22] Galbusera M Noris M Gastoldi S Bresin E Mele C Breno M. An ex vivo test of complement activation on endothelium for individualized Eculizumab therapy in hemolytic uremic syndrome. Am J kidney Dis Off J Natl Kidney Found. (2019) 74:56–72. doi: 10.1053/j.ajkd.2018.11.012, PMID: 30851964

[23] Yuan X Yu J Gerber G Chaturvedi S Cole M Chen H. Ex vivo assays to detect complement activation in complementopathies. Clin Immunol. (2020) 221:108616. doi: 10.1016/j.clim.2020.108616 33148511 PMC8609776

[24] Cavero T Auñón P Caravaca-Fontán F Trujillo H Arjona E Morales E. Thrombotic microangiopathy in patients with malignant hypertension. Nephrol Dial Transplant. (2022) 38:1217–26. doi: 10.1093/ndt/gfac248 36002030

[25] Cavero T Arjona E Soto K Caravaca-Fontán F Rabasco C Bravo L. Severe and malignant hypertension are common in primary atypical hemolytic uremic syndrome. Kidney Int. (2019) 96:995–1004. doi: 10.1016/j.kint.2019.05.014 31420192

[26] Van Den Born BJH Van Montfrans GA. Malignant hypertension. Manag Acute Kidney Probl. (2022):305–16. doi: 10.1007/978-3-540-69441-0_32

[27] Dashe JS Ramin SM Cunningham FG. The long-term consequences of thrombotic microangiopathy (thrombotic thrombocytopenic purpura and hemolytic uremic syndrome) in pregnancy. Obstet Gynecol. (1998) 91:662–8. doi: 10.1097/00006250-199805000-00004, PMID: 9572207

[28] Fakhouri F Roumenina L Provot F Sallée M Caillard S Couzi L. Pregnancy-associated hemolytic uremic syndrome revisited in the era of complement gene mutations. J Am Soc Nephrol. (2010) 21:859–67. doi: 10.1681/ASN.2009070706, PMID: 20203157 PMC2865741

[29] Huerta A Arjona E Portoles J Lopez-Sanchez P Rabasco C Espinosa M. A retrospective study of pregnancy-associated atypical hemolytic uremic syndrome. Kidney Int. (2018) 93:450–9. doi: 10.1016/j.kint.2017.06.022, PMID: 28911789

[30] Morales E Rabasco C Gutierrez E Praga M. A case of thrombotic micro-angiopathy after heart transplantation successfully treated with eculizumab. Transpl Int. (2015) 28:878–80. doi: 10.1111/tri.12551, PMID: 25712140

[31] Morales E Galindo A García L Villalaín C Alonso M Gutiérrez E. Eculizumab in early-stage pregnancy. Kidney Int Rep. (2020) 5:2383–7. doi: 10.1016/j.ekir.2020.09.045, PMID: 33305137 PMC7710878

[32] Gäckler A Schönermarck U Dobronravov V La Manna G Denker A Liu P. Efficacy and safety of the long-acting C5 inhibitor ravulizumab in patients with atypical hemolytic uremic syndrome triggered by pregnancy: a subgroup analysis. BMC Nephrol. (2021) 22:5. doi: 10.1186/s12882-020-02190-0, PMID: 33407224 PMC7786907

[33] Portoles J Huerta A Arjona E Gavela E Agüera M Jiménez C. Characteristics, management and outcomes of atypical haemolytic uraemic syndrome in kidney transplant patients: a retrospective national study. Clin Kidney J. (2021) 14:1173–80. doi: 10.1093/ckj/sfaa096, PMID: 33841863 PMC8023214

[34] Zuber J Le Quintrec M Sberro-Soussan R Loirat C Frémeaux-Bacchi V Legendre C. New insights into postrenal transplant hemolytic uremic syndrome. Nat Rev Nephrol. (2011) 7:23–35. doi: 10.1038/nrneph.2010.155, PMID: 21102542

[35] Le Quintrec M Zuber J Moulin B Kamar N Jablonski M Lionet A. Complement genes strongly predict recurrence and graft outcome in adult renal transplant recipients with atypical hemolytic and uremic syndrome. Am J Transplant. (2013) 13:663–75. doi: 10.1111/ajt.12077, PMID: 23356914

[36] Vande WJ Delmas Y Ardissino G Wang J Kincaid JF Haller H. Improved renal recovery in patients with atypical hemolytic uremic syndrome following rapid initiation of eculizumab treatment. J Nephrol. (2017) 30:127–34. doi: 10.1007/s40620-016-0288-3 26995002 PMC5316393

[37] Ryan M Donato BMK Irish W Gasteyger C L’Italien G Laurence J. Economic impact of early-in-hospital diagnosis and initiation of Eculizumab in atypical Haemolytic Uraemic syndrome. PharmacoEconomics. (2020) 38:307–13. doi: 10.1007/s40273-019-00862-w 31828738 PMC7045788

[38] Licht C Greenbaum LA Muus P Babu S Bedrosian CL Cohen DJ. Efficacy and safety of eculizumab in atypical hemolytic uremic syndrome from 2-year extensions of phase 2 studies. Kidney Int. (2015) 87:1061–73. doi: 10.1038/ki.2014.423, PMID: 25651368 PMC4424817

[39] Barbour T Scully M Ariceta G Cataland S Garlo K Heyne N. Long-term efficacy and safety of the long-acting complement C5 inhibitor Ravulizumab for the treatment of atypical hemolytic uremic syndrome in adults. Kidney Int Rep. (2021) 6:1603–13. doi: 10.1016/j.ekir.2021.03.884, PMID: 34169200 PMC8207473

[40] Claes KJ Massart A Collard L Weekers L Goffin E Pochet JM. Belgian consensus statement on the diagnosis and management of patients with atypical hemolytic uremic syndrome. Acta Clin Belg. (2018) 73:80–9. doi: 10.1080/17843286.2017.1345185, PMID: 29058539

[41] Il CH Jo SK Yoon SS Cho H Kim JS Kim YO. Clinical practice guidelines for the Management of Atypical Hemolytic Uremic Syndrome in Korea. J Korean Med Sci. (2016) 31:1516–28. doi: 10.3346/jkms.2016.31.10.1516, PMID: 27550478 PMC4999392

[42] Schaefer F Ardissino G Ariceta G Fakhouri F Scully M Isbel N. Clinical and genetic predictors of atypical hemolytic uremic syndrome phenotype and outcome. Kidney Int. (2018) 94:408–18. doi: 10.1016/j.kint.2018.02.029, PMID: 29907460

[43] Bouwmeester RN Duineveld C Wijnsma KL Bemelman FJ van der Heijden JW van Wijk JAE. Early Eculizumab withdrawal in patients with atypical hemolytic uremic syndrome in native kidneys is safe and cost-effective: results of the CUREiHUS study. Kidney Int Rep. (2023) 8:91–102. doi: 10.1016/j.ekir.2022.10.013, PMID: 36644349 PMC9832049

[44] Menne J Delmas Y Fakhouri F Licht C Lommelé Å Minetti EE. Outcomes in patients with atypical hemolytic uremic syndrome treated with eculizumab in a long-term observational study. BMC Nephrol. (2019) 20:1–12. doi: 10.1186/s12882-019-1314-1 30971227 PMC6456946

[45] Ariceta G Fakhouri F Sartz L Miller B Nikolaou V Cohen D. Eculizumab discontinuation in atypical haemolytic uraemic syndrome: TMA recurrence risk and renal outcomes. Clin Kidney J. (2021) 14:2075–84. doi: 10.1093/ckj/sfab005, PMID: 35261761 PMC8894930

[46] Macia M de Alvaro MF Dutt T Fehrman I Hadaya K Gasteyger C. Current evidence on the discontinuation of eculizumab in patients with atypical haemolytic uraemic syndrome. Clin Kidney J. (2017) 10:sfw115. doi: 10.1093/ckj/sfw115 PMC5466111 28621343

[47] Olson SR Lu E Sulpizio E Shatzel JJ Rueda JF Deloughery TG. When to stop Eculizumab in complement-mediated thrombotic microangiopathies. Am J Nephrol. (2018) 48:96–107. doi: 10.1159/000492033, PMID: 30110670

[48] Sánchez Chinchilla D Pinto S Hoppe B Adragna M Lopez L Justa Roldan ML. Complement mutations in diacylglycerol kinase-ε-associated atypical hemolytic uremic syndrome. Clin J Am Soc Nephrol. (2014) 9:1611–9. doi: 10.2215/CJN.01640214, PMID: 25135762 PMC4152807

[49] Lemaire M Frémeaux-Bacchi V Schaefer F Choi M Tang WH Le QM. Recessive mutations in DGKE cause atypical hemolytic-uremic syndrome. Nat Genet. (2013) 45:531–6. doi: 10.1038/ng.2590, PMID: 23542698 PMC3719402

[50] Shawky S Safouh H Gamal M Abbas MM Aboul-Enein A Sawai T. Anti-factor H antibodies in Egyptian children with hemolytic uremic syndrome. Int J Nephrol. (2021) 2021:1–8. doi: 10.1155/2021/6904858, PMID: 34840826 PMC8616678

[51] Durey M-AD Sinha A Togarsimalemath SK Bagga A. Anti-complement-factor H-associated glomerulopathies. Nat Rev Nephrol. (2016) 12:563–78. doi: 10.1038/nrneph.2016.99, PMID: 27452363

[52] Raina R Sethi SK Dragon-Durey M-A Khooblall A Sharma D Khandelwal P. Systematic review of atypical hemolytic uremic syndrome biomarkers. Pediatr Nephrol. (2022) 37:1479–93. doi: 10.1007/s00467-022-05451-2, PMID: 35118546

[53] Bresin E Rurali E Caprioli J Sanchez-Corral P Fremeaux-Bacchi V De Cordoba SR. Combined complement gene mutations in atypical hemolytic uremic syndrome influence clinical phenotype. J Am Soc Nephrol. (2013) 24:475–86. doi: 10.1681/ASN.2012090884, PMID: 23431077 PMC3582207

[54] Bruneau S Néel M Roumenina LT Frimat M Laurent L Frémeaux-Bacchi V. Loss of DGKε induces endothelial cell activation and death independently of complement activation. Blood. (2015) 125:1038–46. doi: 10.1182/blood-2014-06-579953, PMID: 25498910

