# Supplementary material for: Recommendations for the individualised management of atypical hemolytic uremic syndrome in adults
Source: Front Med (Lausanne). 2023 Dec 1;10:1264310. doi: 10.3389/fmed.2023.1264310 (PMC10722909; doi:10.3389/fmed.2023.1264310)
Supplement: Supplementary file 1 [file Data_Sheet_1.docx]

Supplementary Material

**Recommendations for the individualised management of atypical hemolytic uremic syndrome in adults**

**Ana Ávila^*^, Mercedes Cao, Mario Espinosa, Joaquín Manrique, Enrique Morales.**

*** Correspondence:** Ana Ávi

la: aaaavilab@gmail.com

# Supplementary Figures and Tables

## Supplementary Figures


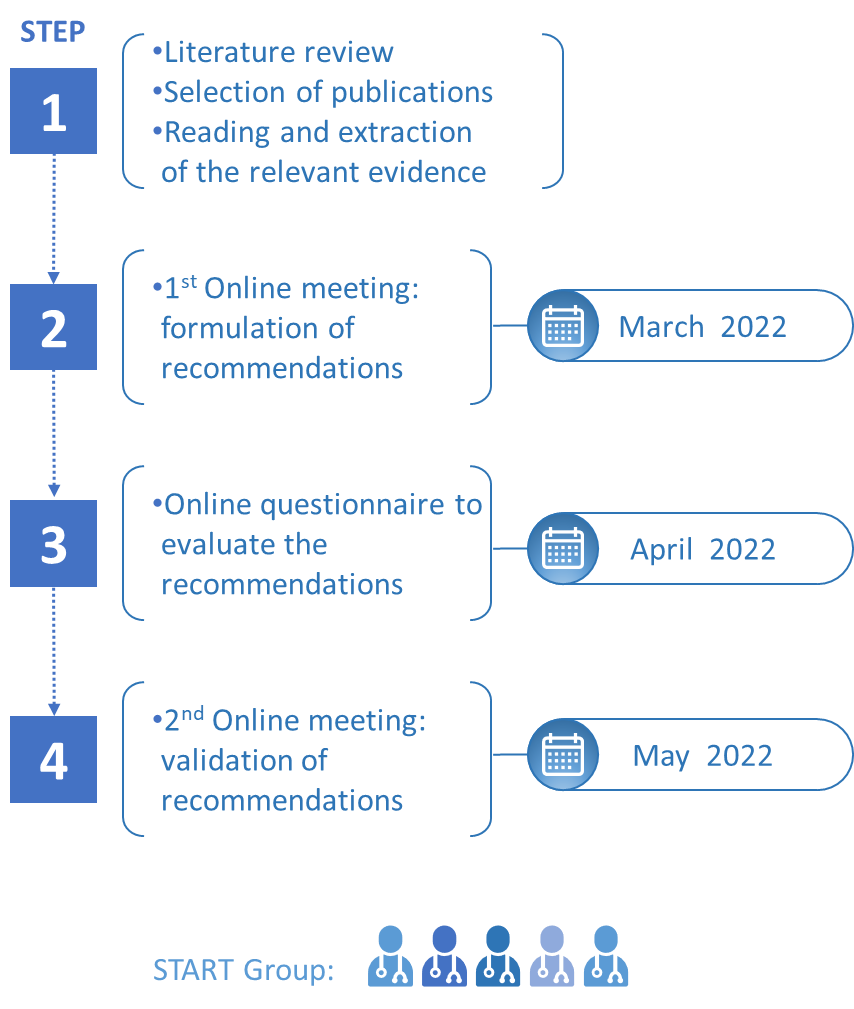


**Supplementary Figure 1.** START consensus workflow chart.


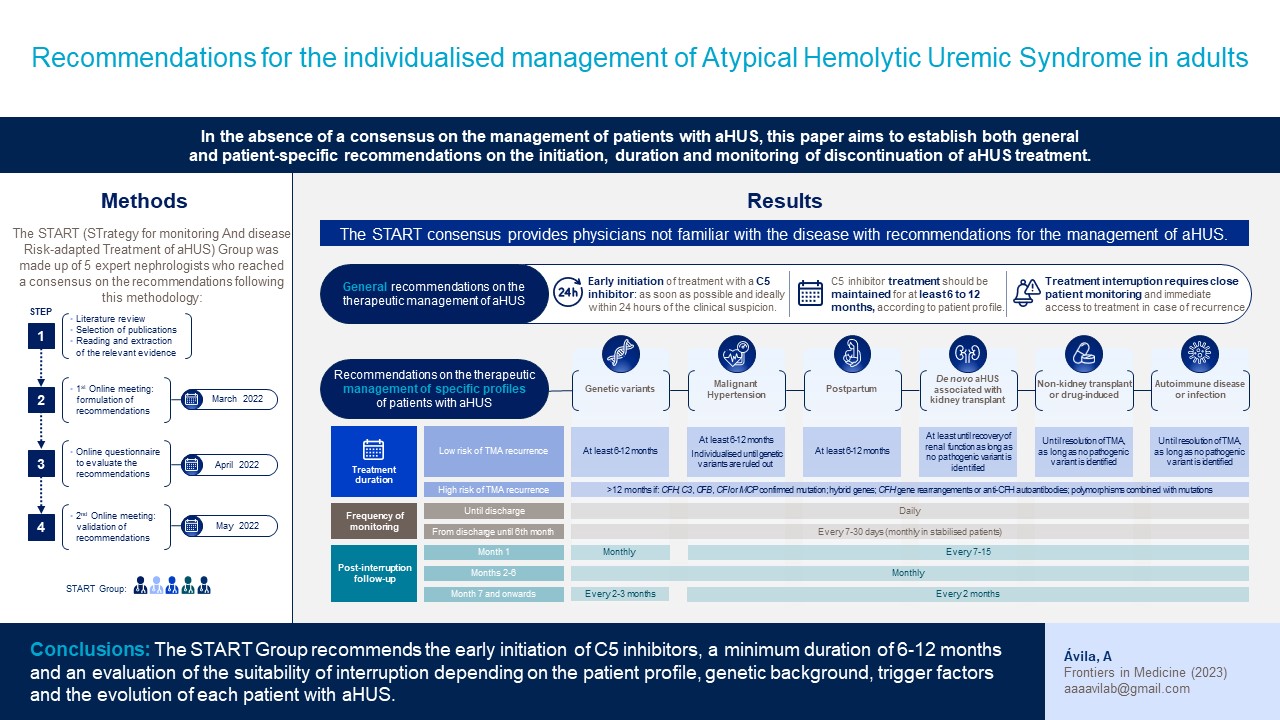


**Supplementary Figure 2.** Visual abstract.

**Supplementary Tables.**

**Supplementary Table 1.** Literature review.

| **Literature** | **DOI** |
| --- | --- |
| Actualización en síndrome hemolítico urémico atípico: diagnóstico y tratamiento. Documento de consenso | DOI: 10.1016/j.nefro.2015.07.005 |
| An international consensus approach to the management of atypical hemolytic uremic syndrome in children | DOI: 10.1007/s00467-015-3076-8 |
| Atypical Hemolytic Uremic Syndrome (aHUS) Patient Journey | European Reference Network |
| Atypical hemolytic uremic syndrome and C3 glomerulopathy: conclusions from a “Kidney Disease: Improving Global Outcomes” (KDIGO) Controversies Conference | DOI: 10.1016/j.kint.2016.10.005 |
| Can eculizumab be discontinued in aHUS? Case report and review of the literature | DOI: 10.1097/MD.0000000000004330 |
| Current evidence on the discontinuation of eculizumab in patients with atypical haemolytic uraemic syndrome | DOI: 10.1093/ckj/sfw115 |
| Defining Treatment Duration in Atypical Hemolytic Uremic Syndrome in Adults: A Clinical and Pathological Approach | https://pubmed.ncbi.nlm.nih.gov/32628650/ |
| Discontinuation of Eculizumab Maintenance Treatment for Atypical Hemolytic Uremic Syndrome: A Report of 10 Cases | DOI: 10.1053/j.ajkd.2014.01.434 |
| Disease Recurrence After Early Discontinuation of Eculizumab in a Patient With Atypical Hemolytic Uremic Syndrome With Complement C3 I1157T Mutation | DOI: 10.1097/MPH.0000000000000505 |
| Economic Impact of Early-in-Hospital Diagnosis and Initiation of Eculizumab in Atypical Haemolytic Uraemic Syndrome | DOI: 10.1007/s40273-019-00862-w |
| Eculizumab and aHUS: to stop or not | DOI: 10.1182/blood.2020010234 |
| Eculizumab discontinuation in atypical hemolytic uremic syndrome: TMA recurrence risk and renal outcomes | DOI: 10.1093/ckj/sfab005 |
| Eculizumab discontinuation in children and adults with atypical haemolytic uremic syndrome: a prospective multicentric study | DOI: 10.1182/blood.2020009280 |
| Eculizumab in atypical hemolytic uremic syndrome: strategies toward restrictive use | DOI: 10.1007/s00467-018-4091-3 |
| Eculizumab interruption in atypical hemolytic uremic syndrome due to shortage: analysis of a Brazilian cohort | DOI: 10.1007/s40620-020-00920-z |
| Eculizumab prevents thrombotic microangiopathy in patients with atypical haemolytic uraemic syndrome in a long-term observational study | DOI: 10.1093/ckj/sfy035 |
| Eculizumab use for kidney transplantation in patients with a diagnosis of atypical hemolytic uremic syndrome | DOI: 10.1016/j.ekir.2018.11.010 |
| Efficacy and safety of eculizumab in atypical hemolytic uremic syndrome from 2-year extensions of phase 2 studies | DOI:10.1038/ki.2014.423 |
| Global initiative for asthma. Global Strategy for astham management anda prevention, 2021. | Available from: www.ginasthma.org |
| How I diagnose and treat atypical hemolytic uremic syndrome. | DOI: 10.1182/blood.2022017860 |
| Improved renal recovery in patients with atypical hemolytic uremic syndrome following rapid initiation of eculizumab treatment | DOI: 10.1007/s40620-016-0288-3 |
| Midterm Outcomes of 12 Renal Transplant Recipients Treated With Eculizumab to Prevent Atypical Hemolytic Syndrome Recurrence | DOI: 10.1097/TP.0000000000001909 |
| Optimal duration of treatment with eculizumab in atypical hemolytic uremic syndrome (aHUS)—a question to be addressed in a scientific way | DOI: 10.1007/s00467-019-4192-7 |
| Outcome of atypical haemolytic uraemic syndrome relapse after eculizumab withdrawal | DOI: 10.1093/ckj/sfaa241 |
| Outcomes in patients with atypical hemolytic uremic syndrome treated with eculizumab in a long-term observational study | DOI: 10.1186/s12882-019-1314-1 |
| Outcomes of a clinician-directed protocol for discontinuation of complement inhibition therapy in atypical hemolytic uremic syndrome | DOI: 10.1182/bloodadvances.2020003175 |
| Outcomes of patients with atypical hemolytic uremic syndrome with native and transplantes kidneys treated with eculizumab: a pooled post hoc analysis | DOI: 10111/tri.13022 |
| Pathogenic Variants in Complement Genes and Risk of Atypical Hemolytic Uremic Syndrome Relapse after Eculizumab Discontinuation | DOI: 10.2215/CJN.06440616 |
| Safety and effectiveness of restrictive eculizumab treatment in atypical haemolytic uremic syndrome | DOI: 10.1093/ndt/gfx196 |
| Subclinical atypical haemolytic uremic syndrome relapse following discontinuation of eculizumab | DOI: 10.1111/nep.12931 |
| Successful therapy switch from eculizumab to mycophenolate mofetil in a girl with DEAP-HUS | DOI: 10.1007/s00467-017-3738-9 |
| Systematic review of atypical hemolytic uremic syndrome biomarkers | DOI: 10.1007/s00467-022-05451-2 |
| Tailored Eculizumab Therapy in the Management of Complement Factor H–Mediated Atypical Hemolytic Uremic Syndrome in an Adult Kidney Transplant Recipient: A Case Report | DOI: 10.1016/j.transproceed.2012.07.141 |
| When to Stop Eculizumab in Complement-Mediated Thrombotic Microangiopathies | DOI: 10.1159/000492033 |
